# Protocol paper: randomized controlled trial of the smart online-to-offline model development for chronic diseases management through digital health in real world setting

**DOI:** 10.1186/s13063-025-08735-8

**Published:** 2025-02-05

**Authors:** Jae Eun Shin, Juho Choi, Heejung Lee, Suk-Won Lee, Juhwan Oh

**Affiliations:** 1 Center for International Studies, Graduate School of International Studies, Yonsei University, Seoul, South Korea; 2 College of Medicine, Seoul National University, Seoul, South Korea; 3 Graduate School of Public Administration, Seoul National University, Seoul, South Korea

**Keywords:** Randomized controlled trial, Online-to-offline service, O2O, Digital health, Chronic disease

## Abstract

**Background:**

Chronic diseases such as diabetes and hypertension pose significant health and economic challenges globally, and South Korea is no exception. Innovative digital health services have the potential to revolutionize chronic disease management by providing patients with real-time, personalized care and empowering them to take an active role in their health. There is a critical need to evaluate the effectiveness of such services. This protocol describes a randomized controlled trial that evaluates the effectiveness of an online-to-offline (O2O) digital healthcare service for patients with chronic diseases, specifically diabetes and hypertension, in Pyeongchang-gun.

**Methods:**

This study presents a comprehensive protocol for the assessment of an online-to-offline (O2O) digital health service model aimed at managing chronic diseases. The study consists of two main groups of participants: those with diabetes and those with hypertension. Participants are randomized into treatment and control arms for each group. The intervention includes personalized digital healthcare support, continuous data monitoring, and online education, with primary care provided by healthcare professionals. To evaluate the primary and secondary outcomes—such as HbA1c, systolic blood pressure, and cholesterol levels—the study applies a range of statistical methods. These include intention-to-treat (ITT) analysis to account for all randomized participants, regression models to estimate the treatment effect, and adjustments for baseline covariates to improve precision. Subgroup analyses will explore variations in treatment effects based on factors such as intervention intensity, comorbidity, and healthcare provider.

**Discussion:**

This protocol outlines a novel approach to evaluating the O2O digital health service model for chronic disease management. It offers insights into the nuanced effects of the intervention, highlighting the potential for tailoring future interventions for maximum benefit. By assessing its real-world effectiveness, this study can inform healthcare policies, expand the application scope of O2O service models, and identify additional chronic diseases that can benefit from digital health services. This research bridges the gap between theory and practice, contributing to evidence-based healthcare decision-making and improving patient outcomes in the era of digital health.

**Trial registration:**

This study is registered at ClinicalTrials.gov of the US National Library of Medicine. The registration number is NCT06150508, and the registered date is 2023–11-29

**Supplementary Information:**

The online version contains supplementary material available at 10.1186/s13063-025-08735-8.

## Administrative information

**Table:** 

Title {1}	Protocol paper: randomized controlled trial of the smart online-to-offline model development for chronic diseases management through digital health in real world setting
Trial registration {2a and 2b}	Registered at ClinicalTrials.gov PRS ClinicalTrials.gov ID: NCT06150508 (Details of this registration can be found at https://clinicaltrials.gov/study/NCT06150508?term=NCT06150508&rank=1)
Protocol version {3}	Version 2.0 (120,523)
Funding {4}	This research was supported by a grant from the Korea Health Promotion R&D Project, funded by the Ministry of Health & Welfare, Republic of Korea [grant number: HS22C0061].
Author details {5a}	1. Center for International Studies, Graduate School of International Studies, Yonsei University; 2. College of Medicine, Seoul National University; 3. College of Medicine, Seoul National University; 4. Graduate School of Public Administration, Seoul National University (Corresponding); 5. College of Medicine, Seoul National University (Corresponding)
Name and contact information for the trial sponsor {5b}	Dr. Yun-chul Hong (ychong1@snu.ac.kr)
Role of sponsor {5c}	Study sponsor and funders have no role or responsibility in study design; collection, management, analysis, and interpretation of data; writing of the report; and the decision to submit the report for publication. They will also have no ultimate authority over any of these activities

## Introduction

### Background and rationale {6a}

Chronic diseases, such as diabetes and hypertension, require ongoing management and monitoring to prevent complications. Telemedicine and digital healthcare services have the potential to improve access to care and self-management for patients by providing patients with “real-time, personalized feedback, and empowering them to take an active role in their care” [1]. American Medical Association has also stated that digital health services can help improve patient outcomes “by providing patients with the tools they need to manage their condition effectively, including personalized treatment plans, self-monitoring tools, and real-time feedback on their progress” [2].

Indeed, previous studies have suggested that digital health services can help improve patient outcomes. A study from India has proven how a mobile message-based diabetes management program improved blood glucose control and reduced HbA1c levels in patients with type 2 diabetes [3]. Similarly, a randomized controlled trial of telemonitoring intervention has proved the improved blood pressure control in patients with hypertension [4]. Digital health interventions, such as telemedicine, have also led to reduced healthcare costs for patients with type 2 diabetes [5]. A recent systematic review examining the effectiveness of telemedicine interventions for patients with chronic diseases found the improved clinical outcomes and patient satisfaction, and also reduced healthcare costs [6]. Yet, the patient outcome of using digital health services has never been rigorously evaluated in South Korea’s setting. It is believed that digital healthcare, embracing various emerging digital technologies, will soon become synonymous with healthcare itself, as digital healthcare is “an unavoidable trend” [7].

In this regard, this randomized controlled trial aims to evaluate the effectiveness of an O2O (online-to-offline) digital healthcare service for patients with chronic diseases (specifically, diabetes and hypertension patients) in Pyeongchang-gun of South Korea. The result of this study may contribute to realize the outcome of mobile-app-based digital health care service for chronic disease patients while providing practical implications for to improve national health care system. The intervention model is designed to tackle the regional challenges in managing chronic diseases while closely aligned to national health policy.

#### Increase in chronic disease patients and medical expenses in Korea

Due to changes in living conditions, rising obesity rates, and an aging population, the number of hypertension and diabetes patients in Korea continues to increase. Recent data from the National Health Insurance Service of Korea shows that the rise in medical expenses is outpacing the increase in the number of chronic disease patients, causing a significant social and economic burden [8]. According to the 2021 National Health Insurance Statistical Yearbook published by the Health Insurance Review and Assessment Service and the National Health Insurance Corporation, the number of chronic disease patients (12 diseases) in 2021 was 20.07 million (an increase of 6.1% from the previous year), and the medical expenses were KRW 39.2109 trillion (an increase of 8.1% from the previous year). By disease, there were 7.06 million hypertension patients and 3.56 million diabetes patients. Medical expenses for hypertension and diabetes were KRW 7.5163 trillion in 2021, which is almost twice (1.95 times) of the expense in 2010 (KRW 3.8420 trillion). It was found that the annual average increase in medical expenses for hypertension was 8.2% and for diabetes was 8.7%.

#### Background of the target area for pilot project, Pyeongchang-gun: increase in chronic disease patients and insufficient management

1. Hypertension

According to the analysis of the National Health Insurance Service’s database, the medical utilization rate due to hypertension in Pyeongchang-gun increased from 21.09% in 2010 to 22.4% in 2019, indicating a gradual rise over the past 10 years [9]. In the meantime, the medical utilization rate for new hypertension patients in Pyeongchang-gun in 2019 was 4.3% of the total population, and it has remained at 4–5% over the previous 10 years. The 1-year control rate for hypertension in Pyeongchang-gun, which refers to the percentage of patients whose systolic blood pressure is below 140 mmHg and diastolic blood pressure is below 90 mmHg during general health examination in the following year, was 62.5%. This rate is about 10% lower than the national average control rate (74.7%), and this trend has been consistent over the past 10 years. The high trend of new medical utilization as well as the relatively low control rate may attribute to insufficient management of chronic diseases.

(2) Diabetes

The medical utilization rate of diabetes patients in Pyeongchang-gun in 2019 (diabetes prevalence-based indicator) was 9.3%, showing a continuous increasing trend over the past 10 years. The medical utilization rate for new diabetes patients (indicator based on new patient incidence rate within a year) was 1.2%, remaining around 1% over the past 10 years.. However, the 1-year control rate for diabetes in Pyeongchang-gun, defined as the percentage of patients whose fasting blood sugar is below 126 mg/dL during their general health examination in the following year, was 39.1% for existing diabetes patients and 47.3% for new diabetes patients as of 2018. This suggests that blood sugar levels are not under control in more than half of those who visit hospitals due to diabetes [9].

#### Promotion of digital health care in Korea

In January 2021, the government announced the “National Health Promotion Plan 2030” to address existing health policy challenges and prepare for future conditions. One of the key action items is the promotion of a smart health city that integrates public health, medical care, and primary care using smart technology. Another important action item is the development of a continuous management system for chronic diseases, such as hypertension, diabetes, and dyslipidemia, aimed at preventing complications and improving healthcare delivery at the primary care level [10].

### Objectives {7}

The objective of this research is to evaluate the effectiveness and efficiency of the O2O service model developed to provide primary healthcare and management of chronic diseases targeting patients residing in Pyeongchang-gun at the local government level.

This randomized controlled trial will help verify the model and provide valuable information on the effectiveness of the O2O digital healthcare service for patients with diabetes and hypertension. If the intervention is found to be effective, it has the potential to improve access to care and self-management for patients, which could lead to improved health outcomes and reduced healthcare costs. The implications from this study can also serve as credible evidence for decision-making for provision of digital health care services in other regions of Korea as well.

## Trial design {8}

This is a superiority randomized controlled trial with restricted, batch, blocked, and individual random assignment. For sampling before randomization, we plan to recruit two target disease groups in Pyeongchang-gun: one group with diabetes patients (including pre-diabetes patients) and another with hypertension patients (including borderline hypertension patients). A total of 1000 participants (500 per group) will be recruited, with priority given to poorly controlled patients during recruitment. Restricted random assignment ensures that the distribution between treatment and control groups remains balanced, while batch random assignment refers to enrolling and randomizing participants in groups over time rather than individually as they come. Blocked random assignment is used to divide participants into blocks based on disease type (diabetes or hypertension), ensuring an even distribution of participants across treatment and control groups within each block. Finally, individual random assignment means that each participant is randomized independently within their block, ensuring an unbiased allocation to either the treatment or control group. These methods help to minimize imbalances and improve the precision of the treatment effect estimates for each condition. Primary mode of sampling is voluntary based, where patients sign up through the advertisement, promotional campaign, or information provided by the physicians. Some patients will be recommended and informed to join this trial by their physicians while promotional/advertising materials will be posted at participating medical centers (i.e., Pyeongchang Public Medical Center and local private clinic, local private medical centers in Pyeongchang) and the Pyeongchang official website. Flyers will also be distributed through voluntary village health support groups, known as the Village Health Committee. In the meantime, for patients who have already diagnosed to have diabetes and hypertension will also be recruited from participating medical centers in Pyeongchang (i.e., local medical centers, Pyeongchang Public Medical Center).

When eligible patients who are willing to participate in this study visits local health center for clinical tests, their data will be periodically sent to the research team to carry out blocked random assignment (block: disease, severity, region). The assignment results are notified to the center/clinic and then to the patients. The allocation ratio is 1:1 for each disease (hypertension and diabetes) as Table 1 illustrates.

**Table 1:** Trial design: sample an allocation plan

	Treatment group	Control group
Hypertension patients	250	250
Diabetes patients	250	250

## Methods: participants, interventions, and outcomes

### Study setting {9}

**Fig. 1:**
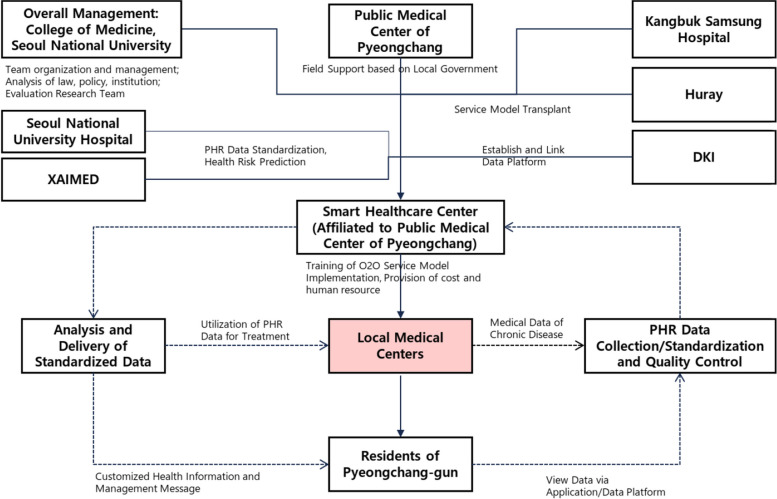
Setting and stakeholders of the intervention

The intervention will take place in Pyeongchang-gun, Gangwon-do, South Korea. (Pyeongchang-gun is a county in the province of Gangwon-do, South Korea. It is in the eastern part of the province with a population of around 43 thousand people estimated in 2020.) The Smart Healthcare Center affiliated to Public Medical Center of Pyeongchang will primarily manage the O2O service by providing necessary education and human resources while receiving and monitoring data from each participant and local medical centers. The overall setting and related stakeholders of the study are illustrated in Fig. 1.

### Eligibility criteria {10}

For participant recruitment, the inclusion criteria is the following:

For both the treatment and control groups, we will enroll adults aged 18 to 70 years with a diagnosis of diabetes (including borderline diabetes) or hypertension (including borderline hypertension). However, we will exercise flexibility in age eligibility, allowing patients above 70 years to participate if they meet the following criteria:

Digital literacy: Participants above 70 years must demonstrate proficiency in digital technology and the ability to effectively use the mobile application “Value Health,” which has been specially developed for this research.

Exclusion criteria are the following:

- Incompatible mobile device: Individuals using older mobile phones that are not compatible with the “Value of Health” application will be excluded.
- Participation in concurrent research: Individuals who are currently participating in another research program involving smart health technology will not be eligible for inclusion in our study.
- Non-acceptance of random assignment or participation: Individuals who do not agree to be randomly assigned to either the treatment or control group or who do not wish to engage in our research program will be excluded from participation.

### Who will take informed consent? {26a}

Smart Healthcare Center of Pyeongchang Public Medical Center will obtain informed consent from all participants prior to enrolment. Informed consent will be received in person.

### Additional consent provisions for collection and use of participant data and biological specimens {26b}

All participants will be provided with sufficient explanation on the purpose and objectives of the study, the items to be collected including personal and sensitive information, details about third-party disclosure of personal information, retention and use period, right to refuse, and potential disadvantages prior to seeking consent. The baseline and regular follow-up medical record as well as the national health screening results will be employed for analysis. Yet, we do not collect biological specimens of the participants.

## Interventions

### Explanation for the choice of comparators {6b}

Patients who are recruited and consented to participate in the study, yet assigned to the control group will be the comparators. Comparators will not be provided with the O2O service, but will be treated as usual (TAU) through the 1st tier health care clinics/centers in Pyeongchang-gun county. TAU typically includes regular visits to their healthcare provider for medication management, routine tests such as HbA1c for diabetes or blood pressure monitoring for hypertension, and home monitoring of fasting blood sugar levels or blood pressure as recommended. This will provide a comparison to the intervention group, which receives additional digital healthcare support.

### Intervention description {11a}

#### Explanation of major stakeholders for intervention

As illustrated in Fig. 1, the overall study (including the design of trial) is managed by Seoul National University College of Medicine while the provision of O2O service and data collection are primarily in charge of Smart Healthcare Center of Pyeongchang.

There are stakeholders responsible for developing and managing the O2O model (specifically the mobile application and data platform), such as Kangbuk Samsung Hospital, Huray, DKI, and Seoul National University Hospital, as well as XAIMED.

- ♦ Kangbuk Samsung Hospital initially developed a prototype for an online service to manage chronic diseases, including diabetes and hypertension, in urban areas.
- ♦ Huray is a digital healthcare company. Collaborating with Kangbuk Samsung Hospital, they designed an online platform to gather patients’ life-log data. This platform provides a disease management intervention program accessible via a mobile application for patients and a webpage for service providers.
- ♦ Seoul National University Hospital is tasked with standardizing patients’ life-log data and creating algorithms to distribute personalized educational content tailored to specific diseases.
- ♦ DKI Technology developed a personal online datastore (POD) that aggregates and safeguards personal health records (PHR). Linking them with the National Health Insurance Service (NHIS).
- ♦ XAIMED developed an explainable AI medical solution that predicts the risk of eye diseases and atherosclerosis using fundus images, which has been integrated into the O2O program.

#### Intervention in O2O service model

Following baseline comprehensive health examinations, each participant’s primary attending physician will develop an individualized care plan. This care plan includes identifying the representative disease group (diabetes, pre-diabetes, hypertension, borderline hypertension) and determining the intensity of care (intensive or standard). In cases where participants have both diabetes and hypertension, they are categorized as diabetes patients. Participants with poorly controlled diseases (systolic blood pressure exceeding 140 or diastolic pressure over 90 in the hypertension group, and HbA1c levels exceeding 6.5% in the diabetes group) are assigned to the intensive care group. However, these classifications can be adjusted as necessary based on the patient’s clinical condition.

The Pyeongchang Smart Healthcare Center continuously monitors life-log data and provides interventions through automated or manual messages. The detailed components of life-log data are described in Supplementary Table 1.

##### Message delivery through the application

Personalized educational material: The content of these messages is determined based on the participant’s baseline evaluation results. They consist of publicly available videos produced by reputable organizations, including academic societies, and are provided via links to YouTube videos.

Health reports: Summary statistics of weekly recorded data (such as blood pressure, blood sugar, medication, exercise, and steps) are provided to the patient through the app.

Manual messages (texted messages): Indicator-based personalized messages are manually transcribed, evaluating the state of health management over the last 2 or 4 weeks based on life-log data. These messages are primarily drafted by the Pyeongchang Smart Healthcare Center, using pre-made message templates created by healthcare experts (doctors, nutritionists, nurses, etc.) suitable for each situation. Messages are sent to patients after final confirmation by their respective primary physicians. Additional messages can be sent as needed during the monitoring process, especially if blood pressure or blood glucose levels are at cautionary or dangerous levels or if data entry is insufficient.

##### Continuous glucose monitor (among the intervention group)

Some diabetes patients in the intervention group will be provided with a continuous glucose monitor (Freestyle Libre). Eligibility for this monitor is determined based on their recorded glucose levels not reaching the normal range in more than half of their recent examinations within the latest 14 days. Patients eligible for the continuous glucose monitor receive notifications via text or phone call from the Smart Healthcare Center. They are required to attach the device for 2 weeks and are advised to self-monitor their glucose variations. During this period, they are strongly encouraged to manage their glucose levels by adapting their usual lifestyle. Some patients may receive the monitor again, if needed, 2 weeks after the detachment of the device.

##### Personal health record (PHR) data

PHR data can be accessed and utilized exclusively by attending physicians with patient consent through the participant’s personal online datastore (POD) web view in the clinic. POD can import health screening results, medical history, and prescription history provided by the National Health Insurance Service (NHIS), which is mandatory for all citizens to enroll in. Additionally, POD contains AI-based retinal image analysis capable of detecting ophthalmologic abnormalities (e.g., glaucoma, diabetic retinopathy) and predicting cardiovascular disease (CVD) mortality risk [11, 12]. It also includes another CVD incidence prediction model developed using national health screening data. With access to this PHR data, attending physicians can provide comprehensive consultations to patients regarding their CVD risk [13].

**Fig. 2:**
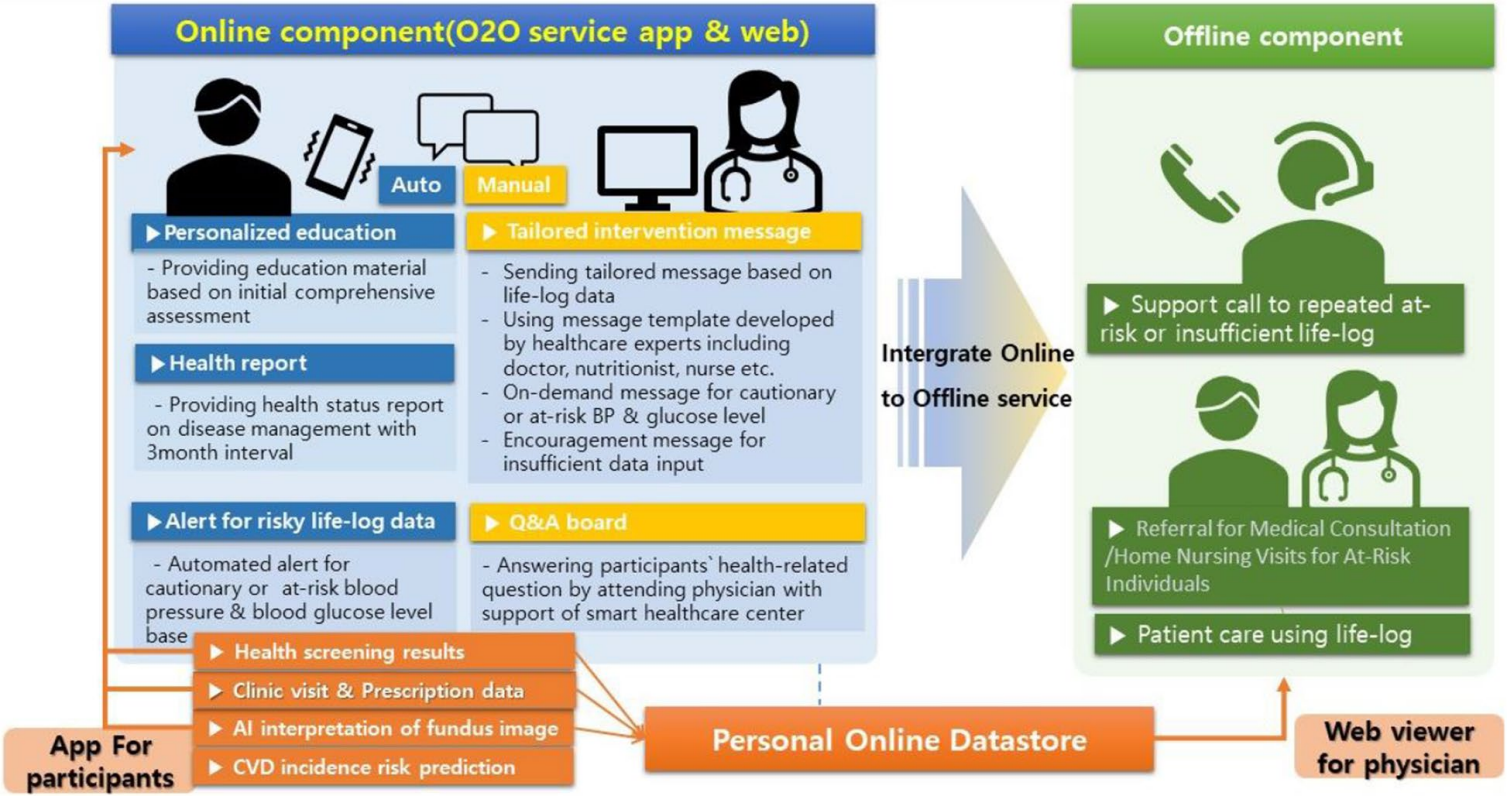
Online and offline components of intervention in the O2O service model and concept of personal online datastore (POD)

The online and offline components of intervention are illustrated in Fig. 2.

#### Control group

Patients in the control group will continue to receive treatment as usual (TAU), following their doctor’s recommendations.

### Criteria for discontinuing or modifying allocated interventions {11b}

Study participants have the right to withdraw their participation from the study at any time if they wish to do so by requesting.

### Strategies to improve adherence to interventions {11c}

Patients will be regularly reminded to use the mobile app to monitor their medical conditions through mobile alarms and notifications. Additionally, the Smart Healthcare Center of Pyeongchang Medical Center will provide more direct reminders, including phone calls and, if necessary, in-person notifications during clinic visits. After patients upload their measurement results, they will receive feedback based on the normal range of the results, displayed using a traffic signal system (e.g., green for normal, yellow for caution, and red for abnormal). If further follow-up is needed, additional text messages will be sent, or phone calls will be made to encourage participation in educational sessions or additional interventions.

### Relevant concomitant care permitted or prohibited during the trial {11d}

Treat of as usual (TAU) is allowed for both control and treatment group. However, we believe that patients’ participation in alternative services will be the most difficult problem to solve during the study. We have identified “participating in another research program involving smart health technology” as one of the exclusion criteria. To prevent concomitant care during the intervention, we will further ensure that participants are properly informed through trained operators. These operators will be carefully educated to instruct participants on the proper utilization of the service and to avoid seeking additional care outside the study protocol.

### Provisions for post-trial care {30}

Not applicable: the intervention does not include any direct health intervention and no possible harm is expected that require post-trial care.

### Outcomes {12}

The primary outcome measures for this study are systolic blood pressure for participants with hypertension and HbA1c for participants with diabetes. The impact on these outcomes will be assessed using the regression-adjusted mean difference and effect size, calculated as the mean difference divided by the standard deviation of the outcome.

Secondary outcome measures include changes in other medical indicators (e.g., fasting blood glucose, cholesterol, triglycerides), medication adherence, body measurements, health behaviors, and healthcare expenditure from baseline. Participation rate and satisfaction will also be collected at the end of the intervention. The full list of data planned to be collected is shown in Table 2.

**Table 2:** Outcome data to be collected

Evaluation criteria		Data	
Effectiveness	Clinical outcome	Major outcome	Systolic blood pressure
			HbA1C
		Secondary outcome	Diastolic blood pressure, fasting blood glucose (FBG), cholesterol, triglyceride (TG), HDL cholesterol, LDL cholesterol
			Body measurement (weight, BMI, waist size) change
			Medication treatment before and after service (Medication Adherence = Duration of Medication Use/Prescription Period)
			Smoking behavior
			Health care expenditure
	Participation outcome	Application utilization level (DAU, WAU, etc.), record rate	
	Satisfaction rate	Satisfaction rate of doctors and care coordinators (interview) Patient satisfaction rate survey	
Prevention of acute complications		Discovery rate of acute complications through healthcare provider monitoring of indicators (such as blood pressure, blood glucose) that can trigger acute complications at interconnected and recorded values	

### Participant timeline {13}

Participant timeline is illustrated in Table 3.

**Table 3:** Participant timeline

Timepoint	2023	2024				2025
	**Q4**	**Q1**	**Q2**	**Q3**	**Q4**	**Q1**
***Advertisement and sampling***	X					
***Enrolment***						
Eligibility screen	X	X				
Informed consent		X				
Allocation		X				
*** Intervention***		X	X	X		
***Data collection***						
Baseline clinical examination		X				
Baseline survey		X				
Life-log data		X	X			
Post-trial clinical examination				X	X	
Post-trial survey				X	X	
***Analysis/reporting***						
Data analysis					X	X
Report					X	X

### Sample size {14}

The sample size for this study was determined based on the requirements of the project sponsor, which set a target of 1000 participants. Given this constraint, we calculated the Minimum Detectable Effect Size (MDES) to ensure the study could still detect meaningful differences between the treatment and control groups with sufficient statistical power. The cohort will be divided into two main groups: participants with diabetes and those with hypertension. Each group will then be randomly allocated into treatment and control subgroups. The calculation of MDES has been carried out separately for the diabetes and hypertension groups, considering the unique characteristics and expected effect sizes for each group. Despite the sample size being predetermined, we are confident that the power analysis shows the study is adequately powered to detect significant effects.

Blocking helps reduce the variance in the outcome variable by improving the balance between treatment and control groups within blocks. Additionally, regression adjustment further reduces the variance by accounting for covariates that explain part of the outcome variability. Both adjustments serve to reduce the standard error, which in turn lowers the Minimum Detectable Effect Size (MDES) in the formula. With a sample of 500 subjects, equally allocated between treatment and control groups (1:1 ratio), a statistical significance level of 5%, and 80% power, the MDES for this study is calculated based on the reduction in standard error through blocking and covariate adjustment. (The constant 2.8 comes from the standard critical values used to calculate the Minimum Detectable Effect Size (MDES) in a two-tailed test with a significance level (alpha) of 5% and power of 80%.):

$$MDES=2.8\left(\sqrt{\frac1{500}+\frac1{500(1-0.5)}}\right)=0.216$$

In this study, the MDES is 0.216. If we assume that the variation in the outcome variable explained by blocked random assignment and covariates is about 25%, the MDES is 0.187. Assuming when 10% of the variation is explained, the MDES is 0.205. The MDES is the smallest change in a metric that an experiment can reliably detect. Cohen suggested that 0.20 is a small effect size, 0.50 is a medium effect size, and 0.80 is a large effect size [14]. Therefore, our study’s MDES demonstrates that we are powered to detect even small effect sizes, which can still be clinically meaningful.

### Recruitment {15}

There are two concurrent recruitment procedures, each with slight distinction. Procedure 1 primarily targets patients previously diagnosed with diabetes, pre-diabetes, hypertension, or borderline hypertension. Procedure 2 targets respondents who have been exposed to our promotion and advertisement efforts. For these respondents who voluntarily asked to enroll, their eligibility will be confirmed when they are either: (1) previously diagnosed with diabetes, borderline diabetes, hypertension, or borderline hypertension (with proof of diagnosis) or (2) newly diagnosed with any of these conditions through the comprehensive health check-up included in this intervention procedure.

## Assignment of interventions: allocation

### Sequence generation {16a}

Participants will be randomized using a computer-generated randomization sequence to eliminate bias and ensure random allocation. This study applies a restricted, batch, blocked, individual random assignment. The randomization sequence is generated periodically based on disease to ensure balanced allocation across treatment and control groups.

### Concealment mechanism {16b}

Mechanism of implementing the allocation sequence is using central telephone and email. The health care staffs and participants will not know the allocation until the research team inform them.

### Implementation {16c}

The research team will generate the allocation sequence and deliver the allocation results to the center and patients directly. The staffs of Smart Healthcare Center of Pyeongchang Public Medical Center will enroll participants and assign participants to interventions.

## Assignment of interventions: blinding

### Who will be blinded {17a}

Once the random assignment is conducted, no blinding will take place for this study.

### Procedure for unblinding if needed {17b}

Not applicable: no blinding is planned for this study.

## Data collection and management

### Plans for assessment and collection of outcomes {18a}

#### Data quality assurance

To ensure the integrity of our survey data, we have implemented several processes to promote data quality. First, we plan to employ duplication risk mitigation measures by cross-referencing participant phone numbers and birth dates. This step helps identify and rectify any duplicate entries, maintaining the accuracy and uniqueness of our dataset.

Second, our assessors will be rigorously trained to conduct surveys effectively. We have developed a comprehensive manual guide to standardize data collection procedures. This guide serves as a valuable resource for our assessors, ensuring consistency and adherence to established protocols.

Furthermore, prior to the commencement of the intervention, the assessors will also undergo a pre-intervention session. During this session, they will receive training on data collection processes, ensuring their familiarity with survey instruments and questionnaires.

Additionally, our Smart Healthcare Center plays a pivotal role as a call center, promptly addressing any issues or queries that may arise during data collection. This continuous support mechanism helps maintain data quality throughout the study.

#### Study instruments

For data collection, we have developed survey questionnaires and interview guides. These instruments have been meticulously crafted, drawing from health outcome indicators aligned with clinical guidelines. This alignment ensures that our data collection tools are both relevant and comprehensive, enabling us to capture essential information related to our study objectives.

Our commitment to data quality and the robustness of our study instruments underpins the reliability and validity of the data collected, ensuring the credibility of our research findings.

#### List of data

Data to be collected for this study can be categorized into “record of random assignment,” “baseline,” “outcome,” “service utilization data,” and “service cost data” and the overall plan for data collection with means of collection is explained in Table 4. More detailed information including means of collection and timepoints for data collection (Supplement Table 1), demographic and health status information (Supplementary Table 2), health record (Supplementary Table 3), and clinical examination data (Supplementary Table 4) is presented in supplement.

**Table 4:** Plan for data collection

Data	Specific data	Means of collection
Record of random assignment	Treatment status indicator, date of random assignment, personal identifier, personal contact information	- Random assignment log
Baseline data	Medical record of hypertension and diabetes, demographic and health status information, clinical examination data, and other health record	- Health screening result and medical test record before intervention - Baseline survey - Interview by the attending physician
Outcome data	Medical record of hypertension and diabetes, life log, other health status and behavior data	- PHR during and after the intervention (from local health centers and smart healthcare centers) - Data log from app and web platform - Medical cost data from National Health Insurance Service - Follow-up survey
Service utilization data	O2O service utilization log	- Service management data and service log
Service cost data	Cost of service provision	- Service accounting data

### Plans to promote participant retention and complete follow-up {18b}

The research team’s strategy to ensure participant retention, minimize contamination, and handle any drop-out is as follows:

- Conduct a pilot random assignment for about a week to identify any issues.
- Continuously monitor the size of treatment group (T) and comparison group (C) to ensure the random assignment remains intact and identify any issues of non-random allocation.
- Control group contamination: The digital healthcare application used in this study is part of a pilot project and is not publicly available for purchase or download. Only authorized participants allocated to the treatment group have access to the application through an online authorization process, minimizing the risk of contamination by the control group accessing the service. However, we will also manage control group contamination with the following measures: (a) Identify contamination caused by participants versus contamination caused by operators. (b) Provide education to operators to prevent contamination. (c) Minimize face-to-face contact between operators and participants to reduce contamination risk. (d) Ensure that random assignment results are directly communicated to participants by the research team. Cross-over management: If a participant from the control group applies for random allocation again or attempts to access treatment services, they will not be removed from the sample. Instead, this will be handled as a no-show adjustment at the analysis stage.

The field operators (i.e., staffs of Smart Healthcare Center and Public Medical Center of Pyeongchang) will regularly follow up with patients to remind them to use the mobile application. Additionally, the patients will also receive automatic notifications via the app and email reminders for service use. If participants request discontinuation, they will be asked to complete the follow-up survey, and their medical records (from medical centers and the National Health Insurance Service) will be collected at the time of discontinuation. For participants who deviate from the study protocol, medical records will be collected once the deviation is identified.

### Data management {19}

The data management plan is the following for each process:

- ◾ Data entry: All data will be entered using electronic data capture (EDC) software. Two independent operators will perform double data entry for accuracy.
- ◾ Coding: A coding manual will be created to ensure consistency in coding. All coded data will be verified by a second operator for accuracy.
- ◾ Security: Access to the data will be password-protected, and only authorized personnel will have access. The data will be stored on a secure server that is regularly backed up.
- ◾ Storage: The data will be stored in an electronic format. The data will be stored on a secure server located within the research institution.
- ◾ Data quality processes: Range checks will be performed to ensure that data values fall within acceptable ranges. Data cleaning procedures will be performed to correct any discrepancies or missing data. The final dataset will be verified for completeness and accuracy by the research team before analysis.
  ◾ Backup and archiving: The data will be regularly backed up to ensure its integrity in case of system failure or loss. A copy of the final dataset will be archived in a secure location for future reference. According to Article 15 of the Enforcement Rules of the Bioethics and Safety Act, all research-related records and data will be archived for 3 years from the end of the research.
- ◾ Data disposal: Seoul National University, an institution that is in charge of retention, will dispose of any data and document containing personal information in accordance with Article 16 of Enforcement Decree of the Personal Information Protection Act after 3 years of archiving.

### Confidentiality {27}

We will collect personal information about potential and enrolled participants through informed consent forms, medical records, and questionnaires. We will ensure that all personal information is securely stored and accessed only by authorized personnel. We will share personal information only with the research team and regulatory agencies, and we will do so through password-protected access or encryption to ensure confidentiality.

The specific protocols are as follows:

- ◾ The participant is provided with sufficient explanation and, if voluntarily agreed, signs a written consent form to participate in the research.
- ◾ Confidentiality will be maintained for information that can identify the personal information.
- ◾ Information can be provided if the participant inquires about the test results.
- ◾ The participant’s data file will be stored on the server of the data management personnel. This server is accessible only to the principal investigator, research staff, and co-researchers, and the file is encrypted. Therefore, access is not possible except for those who participated in this research.
- ◾ The data file collected during the research process will be provided to a third party (National Health Smart Management Research and Development Division under the Ministry of Health and Welfare, Government of South Korea) and submitted to the data platform (big data and personal data repository) established by the division. In this process, personal information is protected by applying the integrated authentication system and DID (Decentralized Identifier, a distributed identification technology based on blockchain technology) system developed by the division.

### Plans for collection, laboratory evaluation, and storage of biological specimens for genetic or molecular analysis in this trial/future use {33}

Not applicable: this study does not collect any biological specimens.

## Statistical methods

### Statistical methods for primary and secondary outcomes {20a}

We will generate descriptive summaries by comparing key characteristics and pre-treatment outcomes between the treatment and control groups.

We will use regression adjustment to estimate both the intention-to-treat (ITT) effect and the treatment-on-the-treated (TOT) effect for analyzing primary and secondary outcomes. Statistical significance will be assessed using the coefficient estimate from the regression-adjusted model, with significance levels reported for each estimate. Effect sizes will be calculated by dividing the regression-adjusted impact estimate by the standard deviation of the outcome variable in the control group. In addition to analyzing the entire sample, we will conduct separate analyses for participants with diabetes and those with hypertension to evaluate the differential impacts of the intervention on these two groups.

The impact on these outcomes will be evaluated using ① the regression-adjusted mean difference between groups and ② the effect size, which will be calculated as the mean difference divided by the standard deviation of the outcome. Regarding the time point for evaluating the primary outcomes, as the random assignment dates vary between cohorts, we will estimate the impact using outcome data collected at the same elapsed time relative to the random assignment date for all participants.

We will also conduct a longitudinal or repeated measures regression analysis to assess the effects over time. Understanding the occurrence of program effects and how these effects evolve over time is crucial for this experimental study. Analyzing the temporal trajectory of program effects is essential, as the timing of impact and the nature of the intervention can result in varying patterns of change depending on the severity of the disease and the behavioral patterns of the participants.

In randomized experiments, the comparability between randomly assigned treatment and control groups remains stable over time. Therefore, analyzing time-series changes in program effects will be done by using a longitudinal model that measures the outcome variables at multiple time points. This model allows for repeated measurements to estimate program effects over time. It is important to note that in this analysis, “time” will be treated as a relative measure, calculated from each participant’s random assignment date, rather than an absolute calendar time.

Furthermore, in cases where outcomes correspond to both disease blocks (i.e., outcomes related to both diabetes and hypertension), we will pool the samples from each block and conduct pooled regression with block fixed effects. This approach will help reduce the standard errors of the estimated effects while accounting for block-level variations. This addition allows us to improve the precision of our estimates for outcomes that span across both disease blocks (i.e., diabetes and hypertension), while maintaining the integrity of the block design.

### Interim analyses {21b}

The intervention is 6 months to 9 months long, and we do not plan to carry out any interim analyses.

### Methods for additional analyses (e.g., subgroup analyses) {20b}

We plan several subgroup analyses to gain insights into the varied effects of our intervention across different participant profiles. This will be done by including an interaction term for two variables representing relevant subgroups intervention intensity (one vs. the other) in the analysis model to evaluate the differential impact of the intervention strategies on these two groups:

1. General intervention group versus intensive intervention group
2. Participants with coexisting diabetes and hypertension
3. Participants group by responsible healthcare provider
4. Borderline disease group vs. regular disease group
5. Dosage effect analysis

In addition to these subgroup analyses, we will conduct a participation analysis (or per protocol analysis) by comparing the treatment’s effect on participants who adhered well to the protocol within the treatment group to those whose adherence was low. Adherence metrics, such as frequency and consistency of data input, app log-in activity, and response rates to automated and manual messages, will be used. Interaction terms for adherence levels will be included in the model to determine how adherence influences the intervention’s effectiveness, providing insights into the importance of participant engagement in achieving optimal outcomes.

### Methods in analysis to handle protocol non-adherence and any statistical methods to handle missing data {20c}

Defiers in the treatment group: In the event that there is non-adherence to the protocol, particularly if there are individuals who defy the assigned treatment, we will estimate the Local Average Treatment Effect (LATE). Since the control group will not be provided with the application, there is no possibility of “defiers” in the control group. Therefore, we will simply divide the ITT with the rate of compliers, as illustrated below.

$$LATE=\frac{ITT}{Rate\;of\;Compliers\;(in\;Treatment\;Group)}$$

To address missing data for variables of interest, we will use a missing-dummy approach, specifically employing dummy variable adjustment. This method involves creating dummy variables to account for missing data and subsequently including these dummy variables in our analysis as covariates. This approach allows us to compensate for missing data and maintain the completeness of our analysis while minimizing the impact of missing observations on our results.

### Plans to give access to the full protocol, participant-level data, and statistical code {31c}

Data will not be available for public a few years until Ministry of Health (MOH) of Korea finalizes preparation including deidentifying via MOH-based publicly available data set for general health research. The full study protocol and statistical code can be made available upon reasonable request to the corresponding author.

## Oversight and monitoring

### Composition of the coordinating center and trial steering committee {5d}

**Fig. 3:**
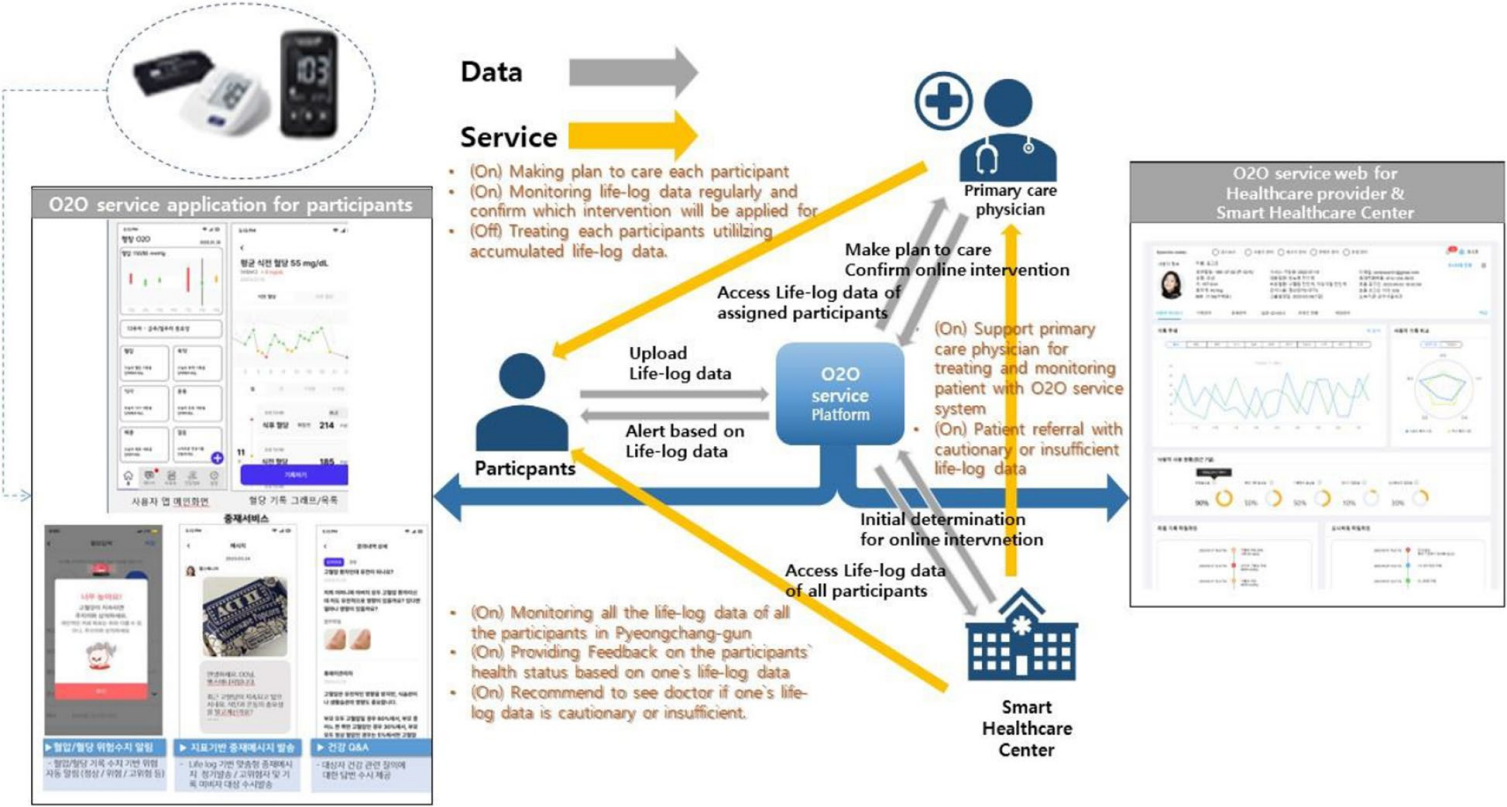
The key players of O2O service model and the data flow among them

Coordinating center (Smart Healthcare Center) responsibilities: The Smart Healthcare Center, operating under the Public Health Center (PHC) of Pyeongchang-gun, serves as the coordinating center for the trial. Its primary role is to monitor participants’ life-log data via the O2O service web page, oversee the automated dispatch of intervention messages, and manage educational content based on the collected data. Additionally, the center drafts manual intervention messages for review and approval by the participants’ primary care physicians. The center is responsible for ensuring the smooth execution of the trial by providing human and material resources and facilitating communication among key stakeholders. The key stakeholders of O2O service model and the data flow among them are illustrated in Fig. 3.

Steering Committee: The Trial Steering Committee, represented by the Pyeongchang Medical Center Board of Directors, provides strategic oversight and governance for the trial. The committee meets monthly to review the trial’s progress, make high-level decisions, and ensure that the study is conducted according to the established protocols. The TSC plays a critical role in overseeing the overall execution of the trial, addressing challenges, and guiding the study to its successful completion.

### Composition of the data monitoring committee, its role and reporting structure {21a}

The Data Monitoring Committee (DMC) is composed of an independent group of experts who are not involved in the trial evaluation. The committee includes medical professional, statistical expert, ICT specialist, ethics and legal expert, as well as a patient representative. This independent oversight ensures the integrity and impartiality of the data monitoring process throughout the trial.

### Adverse event reporting and harms {22}

The likelihood of adverse events or unintended effects related to trial interventions or trial conduct is not anticipated to be high. However, in the event that such occurrences do arise, our response protocol is well-established. Any solicited or spontaneously reported adverse events will be promptly reported to the principal investigator (PI) overseeing the trial. Simultaneously, these events will be reported to the Seoul National University Hospital (SNUH) Institutional Review Board (IRB) Committee, the regulatory body responsible for overseeing the ethical and safety aspects of the trial. Upon notification of an adverse event or unintended effect, relevant and appropriate actions will be taken in response. These actions may include further investigation, adjustments to trial conduct, or any necessary modifications to ensure participant safety and the integrity of the trial.

### Frequency and plans for auditing trial conduct {23}

We do not have a plan for auditing trial conduct.

### Plans for communicating important protocol amendments to relevant parties (e.g., trial participants, ethical committees) {25}

Any changes to the protocol will require approval from the principal investigator, the Steering Committee, the Public Medical Center of Pyeongchang, and the funder, the Korea Health Industry Development Institute. Major amendments will also be communicated to the ethics committee for approval, while minor changes (such as administrative updates or clarifications) do not require ethics approval. Relevant parties, including trial participants and other stakeholders, will be informed of important amendments as necessary.

## Dissemination plans {31a}

Results of the study will be reported to the funding organization and government. Further, results of the study will be disseminated through peer-reviewed publications and presentations at scientific conferences. When the effect of intervention is confirmed from this research, the control group will be provided with the same intervention (O2O service).

## Discussion

This study marks the pioneering implementation of an O2O (online-to-offline) service provision model in collaboration with the local government. We anticipate several valuable contributions to the field through this study. Firstly, by empirically evaluating the O2O service model, we aim to expand its application scope for preventing complications of chronic diseases. The improvement in usability and functionality based on empirical results will be invaluable in making this service accessible to a broader population. Secondly, we aspire to promote evidence-based nationwide expansion of this model, making it accessible to individuals in other areas who may benefit from it. This expansion aims to make the benefits of digital health services available to a wider population. Lastly, the study may also help identify additional chronic diseases that could be effectively managed using digital health services. By closely monitoring how the O2O service model impacts patients with diabetes and hypertension, we will gain valuable insights into the types of chronic disease management challenges that can be addressed with digital health tools. This approach could be applied to other chronic conditions that require regular monitoring, medication adherence, and lifestyle changes—such as cardiovascular disease, respiratory conditions, or obesity—helping to expand the potential applications of O2O models. Through this evaluation, we will explore whether similar benefits, such as improved self-management and reduced complications, can be extended to a wider range of chronic conditions.

In the meantime, it presents several practical and operational challenges and opportunities that merit discussion as follows.

### Introduction of a novel service structure

One of the foremost operational challenges involved the development and introduction of the O2O service provision structure. This required careful planning and coordination between our research team, healthcare professionals, and the local government’s Public Health Center. The process included the establishment of service guidelines and protocols, which will be essential for the successful application of this model in local communities.

### Increase awareness and uptake of the service

Increasing awareness of the O2O service among the target population is another operational hurdle we addressed. Since this service model represents a novel approach to chronic disease management, raising awareness and encouraging participation was vital. This challenge has led to the development of strategies to engage potential users and educate them about the benefits of the O2O service.

### Institutionalization of the service

Our study has ambitious goals, including the institutionalization and enhancement of O2O service models for chronic disease management. Based on this trial, we plan to develop an activation plan that bridges policy with O2O services for chronic disease management. This plan will be instrumental in aligning government health policy with digital health and primary care, creating a synergy that benefits the community.

### Generalizability of the study findings

We acknowledge that the generalizability of our findings may be influenced by the specific characteristics of our voluntary sample. Our study sample, drawn from Pyeongchang-gun, primarily consists of individuals with diabetes and hypertension. As this population is from a rural setting and may exhibit certain age and gender distribution and digital literacy level different from broader populations, particularly in urban areas or among those less comfortable with digital health tools. We are collecting comprehensive socioeconomic data alongside medical data, which will allow us to analyze the characteristics of our sample and examine the types of populations to which these findings can be generalized. Additionally, we will conduct an analysis of the characteristics of compliers in the case of LATE (Local Average Treatment Effect) estimates. These analyses will help to further determine the generalizability of our results.

In conclusion, our efforts are geared towards institutionalizing the O2O service model and making it accessible to a broader population, thereby contributing to the advancement of digital health and primary care in the realm of chronic disease management.

## Trial status

Protocol version 2.0 dated 5 December 2023. The advertising for participation has begun in December 2023, and the recruitment process is slowly taking place concurrently with patients signing up. Finalization of the recruitment/sampling and actual enrolment (i.e., receipt of informed consent, random assignment) will begin in January 2024 as explained in the “Participant timeline” section.

## Consent for publication {32}

Please find the consent form (translated English) in the supplementary materials.

Competing interests {28}.

The authors declare that they have no competing interests.

## Supplementary Information

Supplementary Material 1.

Supplementary Material 2.

## Data Availability

The trial data is accessible only to the principal investigator, research staff, and co-researchers, and the file is encrypted. The data file collected during the research process will be provided to National Health Smart Management Research and Development Division under the Ministry of Health and Welfare, and submitted to the data platform (big data and personal data repository) established by the division. In this process, personal information is protected by applying the integrated authentication system and DID (Decentralized Identifier, a distributed identification technology based on blockchain technology) system developed by the division. Trial data will be available upon request to National Health Smart Management Research and Development Division.

## Institution

PMC: Public Medical Center
SHC: Smart Healthcare Center
NHIS: National Health Insurance Service

## Clinical information

FBG: Fasting blood glucose
TG: Triglyceride
HDL: High-density lipoprotein
LDL: Low-density lipoprotein
BMI: Body mass index
eGFR: Estimated glomerular filtration rate

## Service information

O2O: Online-to-offline
PHR: Personal health record
POD: Personal online storage
DAU: Daily active users
WAU: Weekly active user
EDC: Electronic data capture

## Methodology and statistics

TAU: Treatment as usual
MDES: Minimum Detectable Effect Size
IC: Informed consent
BLD: Baseline data collection
RA: Random assignment
ITT: Intention-to-treat
LATE: Local Average Treatment Effect
DMC: Data Monitoring Committee
