# Supplementary material for: Protocol paper: randomized controlled trial of the smart online-to-offline model development for chronic diseases management through digital health in real world setting
Source: Trials. 2025 Feb 5;26:40. doi: 10.1186/s13063-025-08735-8 (PMC11800493; doi:10.1186/s13063-025-08735-8)
Supplement: Supplementary file 1 — Supplementary Material 1. [file 13063_2025_8735_MOESM1_ESM.docx]

**Supplement 1**

Table 1. Life-log data (Mobile App or Web platform)

Table 2. Demographic and health status information

Table 3. Health Record: Information to be collected by attending physicians

Table 4. Clinical examination data

**[Table 1] Life-log data (Mobile App or Web platform)**

| **Data** | **Specific data** | **Means of collection; Timepoint** |
| --- | --- | --- |
| Blood glucose | Blood glucose Level, Timing of Blood glucose Measurement, Symptoms, Notes | Connected to a glucose meter (manual input possible)  Intensive Management Group – Recommended to record at least twice a week  Standard Management Group – Recommended to record at least once a week |
| Blood Pressure | Systolic Blood Pressure, Diastolic Blood Pressure, Symptoms, Notes, Time of Recording, Pulse, Pulse Pressure | Connected to a blood pressure monitor (manual input possible)  Intensive Management Group – Recommended to record at least twice a week  Standard Management Group – Recommended to record at least once a week |
| Weight | Waist Circumference, Body Fat, Weight | Manual input,  Recommended to input as needed |
| Medication | Type of Medication, Type of Insulin, Dosage, Medication Taken, Injection Site, Time of Recording, Notes | Manual input,  Those on medication are recommended to record daily |
| Exercise | Exercise Duration, Time of Recording, Type of Exercise | manual input,  recommended to input as needed |
| Daily Steps: | Date of Recording, Step Count, Distance, Calories Burned | Synchronized with mobile phone,  inputs as needed |
| Diet | Date of Recording, Meal Time, Type of Meal, Portion Size, Food Pictures | manual input,  recommended to input as needed |

**[Table 2] Demographic and health status information**

| **Data** | **Specific data** | **Means of collection; Timepoint** |
| --- | --- | --- |
| General Information: | Name, Age, Gender, Date of Birth, Contact Information, Address | Survey,  Once at baseline |
| Demographic Characteristics | Education Level, Marital Status, Monthly Income, Occupation | Survey,  Once at baseline |
| Living Environment | Type of House Building, Duration of Residence, Number of Family Members, Presence of Pets | Survey,  Once at baseline |
| Family and Medical History | Presence of a family history of Hypertension/Diabetes/Brain Disorders/Cardiac Diseases, History of Medication for Hypertension/Diabetes/Dyslipidemia | Survey,  Once at baseline |
| Lifestyle Habits | Smoking status, Duration of Smoking, Alcohol Consumption, Frequency of Drinking, Average Daily Alcohol Consumption, Sleep-Related Symptoms (snoring, etc.), Sleep Duration, Engagement in Aerobic Exercise, Weekly Exercise Frequency, Average Daily Exercise Duration | Survey,  Once at baseline |
| Dietary Habits | Number and Amount of Daily Meals, Meal Times, Nutritional Imbalance (Selective Eating, Skipping Meals, Overeating), Eating Out Frequency, Snack Intake (Frequency, Time, Type), Late Night Snack Intake, Frequency of Consuming Fried/Instant/Fast Food, Frequency of Consuming Food Items (Grains/Legumes/Fruits/Vegetables/Dairy Products) | Survey(,  Once each at baseline and endline(Total 2 times) |
| Mental health | Joy or interest, Depression or despair | Survey,  Once each at baseline and endline(Total 2 times) |
| Self-care Competence | Evaluation of One’s Health Status, Practice of Disease Management, Establishment of Disease Management Plan, etc. | Survey,  Once each at baseline and endline(Total 2 times) |
| Motivation  for disease management | Having a Regular Plan for Blood Pressure/Blood Sugar Measurement, Having a Regular Medication Intake Plan, Having a Regular Exercise and Health Management Practice Plan | Survey,  Once each at baseline and endline(Total 2 times) |
| Satisfaction Level | Satisfaction with Intervention Messages and Educational Content, Appropriateness of App Content, Satisfying/Dissatisfying Features, Overall Satisfaction with the Program | Survey,  Once at endline |

**[Table 3] Health Record: Information to be collected by attending physicians**

| **Data** | **Specific data** | **Means of collection; Timepoint** |
| --- | --- | --- |
| Medical history | Diagnosis of Hypertension/Diabetes, Date of First Onset | Interview,  Once at baseline |
| Medication | Whether currently taking medication, Presence of side effects from prescribed medication, Patient medication adherence (number of days medication was not taken in the past 100 days) |  |
| Disease Assessment | Whether current blood pressure/blood glucose levels are normal, Suspicion of secondary hypertension |  |
| History of  Other Diseases | Brain disorders, Heart diseases, Vascular diseases, Kidney diseases, Eye diseases, Neurological disorders, Foot lesions, Other diagnoses and treatments |  |
| Other Medication Histories | steroids/non-steroids, immunosuppressants, anticoagulants, etc. |  |

|  |  |  |
| --- | --- | --- |
|  |  |  |
|  |  |  |
|  |  |  |
|  |  |  |
|  |  |  |
|  |  |  |
|  |  |  |

**[Table 4] Clinical examination data**

| **Data** | **Specific data** | **Means of collection ;Timepoint** |
| --- | --- | --- |
| Physical examination | Height, Weight, BMI, Waist Circumference, Blood Pressure, and Pulse Rate | Once each at baseline and endline  (Total 2 times) |
| Clinical Test | - Blood Test: Glycated Hemoglobin, Fasting Blood Sugar (Health Risk Prediction Variable of Seoul National University Hospital), Total Cholesterol, Triglycerides, LDL Cholesterol, HDL Cholesterol, Serum Creatinine, eGFR, Sodium, Potassium, Hemoglobin  - Urine Test: Urine Albumin/Creatinine Ratio, Hematuria, Proteinuria, etc.  - Fundus Examination (XAIMED) |  |
| Note: These items can refer to medical records or health examination results (especially the general health screening conducted by NHIS) from around three months. | | |
