# Supplementary material for: Protocol paper: randomized controlled trial of the smart online-to-offline model development for chronic diseases management through digital health in real world setting
Source: Trials. 2025 Feb 5;26:40. doi: 10.1186/s13063-025-08735-8 (PMC11800493; doi:10.1186/s13063-025-08735-8)
Supplement: Supplementary file 2 — Supplementary Material 2. [file 13063_2025_8735_MOESM2_ESM.docx]

**Explanation for Research Participants**

1. **Research Title**

Development and Demonstration of an O2O Service Model for Chronic Disease Management Based on Digital Health

1. **Principal Investigator**

Principal Investigator: Professor Oh Ju-hwan, Department of Medicine, Seoul National University College of Medicine

Performing Institution: Seoul National University College of Medicine Research

Commissioned by: Korea Health Industry Development Institute

1. **Background and Purpose of the Research**

The number of patients with chronic diseases is increasing daily, and the associated medical expenses are becoming a burden on society as a whole. However, Pyeongchang County showed somewhat insufficient levels in terms of health management behavior compared to the national average, and the management of chronic disease patients was also found to be inadequate. To manage these chronic diseases, the government is piloting a chronic disease management system. However, the chronic disease management system has limitations in providing incentives to adopt developing smart healthcare technologies and has not developed into a system where the community medical system can cooperate comprehensively.

Therefore, this study aims to establish an organic cooperation system between private medical institutions in the community and the Pyeongchang County Health Medical Center to manage chronic diseases for Pyeongchang County residents. We plan to actively introduce smart healthcare technology to develop and demonstrate an Offline to Online (O2O) service model that encompasses both online and offline services.

1. **Number of Research Participants and Participation Period**

Pyeongchang County residents who showed blood pressure or blood sugar levels outside the normal range in the National Health Insurance Service general health examination results within the past two years, and who meet the following conditions:

- Those previously diagnosed with hypertension or diabetes
- Those who are not previously diagnosed but are in the pre-hypertension or pre-diabetes stage

The research participation period is 6 months from the start date of participation.

1. **Clinical Research Procedures and Methods**

Research participants will receive periodic treatment for chronic diseases (hypertension or diabetes) through cooperating medical institutions in Pyeongchang County. Participants will be provided with smart devices (glucose meter, blood pressure monitor, scale) for self-measurement according to their condition, and a mobile service application developed by the research team will be installed on the participant's own mobile phone. Using the installed application and provided self-measurement devices, health information from daily life such as blood sugar, blood pressure, weight, medication, meals, and physical activity will be automatically collected or partially manually recorded.

The research team will analyze health-related information generated from medical institutions and daily life to predict individual participants' health risks and provide customized health management education content online. The research team will also monitor this health information and guide online or telephone counseling or medical treatment for those with abnormal findings.

The research team will conduct surveys on research participants at the start, middle, and end points of participation. Survey items include demographic information, health and chronic disease-related information, lifestyle habits, family history, occupational history, digital literacy, and service model satisfaction.

1. **Anticipated Side Effects, Risks, and Discomforts for Research Subjects**

This study is research on a chronic disease management service delivery model and does not plan any invasive procedures such as medical techniques or medication. Therefore, this study is considered to have minimal risk, and no side effects, risks, or discomforts are anticipated for patients.

1. **Anticipated Benefits for Research Subjects**

To manage chronic diseases, in addition to temporary contact with medical staff, means are needed to objectively evaluate and intervene in health behaviors in daily life. This study utilizes comprehensive personal health information generated from medical institutions and daily life, allowing participants to experience a higher level of chronic disease management. Through this, we expect to prevent complications from chronic diseases and extend healthy life expectancy.

1. **Research Participation Costs and Compensation for Losses**

All costs related to the entire process of research participation are provided from the research fund, and you will not be charged any costs. Also, if you participate in this study, you will receive smart devices for self-measurement related to chronic diseases (worth about 100,000 won). However, you must bear the cost of medical treatment and medications for chronic diseases as before. There are no anticipated risks from participating in this study, and therefore, there is no plan for loss compensation.

1. **Voluntary Participation and Withdrawal of Consent**

You can decide to participate in this clinical study of your own free will. You can express your intention to refuse participation in this study at any time, and there will be no disadvantages or liability for compensation to you at that time. If you withdraw your consent during the study, the data collected up to that point will be discarded.

1. **Matters Concerning the Preservation and Disposal of Human-derived Materials**

This study does not plan to obtain human-derived materials from participants.

1. **Matters Concerning the Provision of Human-derived Materials and Genetic Information Obtained from Them**

This study does not plan to obtain human-derived materials from participants.

1. **Matters Concerning the Disposal or Transfer of Human-derived Materials in Case of Abnormal Termination of Human-derived Material Research**

This study does not plan to obtain human-derived materials from participants.

1. **Matters Concerning the Retention Period and Information Disclosure of Human-derived Material Research Results**

This study does not plan to obtain human-derived materials from participants.

1. **Matters Concerning Personal Information Protection and Provision of Personal Information**

Records containing personal information that can identify the subjects participating in this study will be kept confidential, and if the research results are published, the identity of the subjects will be protected as confidential.

During the clinical trial and after the research trial is completed, monitors of the clinical research, inspectors, the IRB, and the Minister of Health and Welfare may review the subject's research records within the scope of protecting the confidentiality of the subject's identity to verify the procedures and data quality of the research in accordance with relevant laws. This access to data will be permitted by the consent form signed by the subject or the subject's representative.

Information collected during the research process is planned to be provided to a third party (National Health Smart Management R&D Project Group under the Ministry of Health and Welfare) and will be submitted to the data platform (big data and personal data repository) established by this project group. In the process of submitting data, personal information will be protected by applying the integrated authentication system and DID (Decentralized Identifier, blockchain technology-based distributed identity verification technology) system developed by this project group.

1. **Contact Information**

The principal investigator of this study is Professor Oh Ju-hwan of the Department of Medicine, Seoul National University College of Medicine. For inquiries about this study, please contact the Department of Medicine, Seoul National University College of Medicine (Phone: 02-740-8340). If you have any problems, concerns, or questions about the rights of research subjects, you can request assistance from the Seoul National University College of Medicine/Seoul National University Hospital Institutional Review Board (IRB) contact (02-2072-0694) or the Clinical Research Ethics Center contact (02-2072-3509).

**Informed Consent Form**

1. I have received a verbal explanation about the clinical research, read the above research explanation, and have sufficiently discussed this research with the responsible researcher.
2. I have been informed about the risks and benefits of the research and have received satisfactory answers to my questions.
3. I voluntarily consent to participate in this research.
4. I understand that I can refuse to participate in the research or withdraw my participation at any time without affecting my subsequent treatment, and that this decision will not cause me any harm.
5. By signing this explanation and consent form, I agree that my personal information may be collected and processed by the researcher within the scope permitted by current laws and regulations for medical research purposes.
6. I understand that I will receive a copy of the research explanation and consent form.

|  |  |  |  |  |
| --- | --- | --- | --- | --- |
| Research Subject's Name |  | Signature |  | Date(Year/Month/Day) |
|  |  |  |  |  |
| Researcher's Name |  | Signature |  | Date(Year/Month/Day) |
|  |  |  |  |  |
| Legal Representative's Name |  | Signature |  | Date(Year/Month/Day) |
|  |  |  |  |  |
| (Relationship between subject and representative) |  | (Reason for representative consent) |  |  |

**Authorization for Use and Disclosure of**

**Personal Health Information**

**1. I have received a detailed explanation of the content of the "Development and Demonstration of an O2O Service Model for Chronic Disease Management Based on Digital Health" and this consent form, fully understand its contents, and agree to participate in the research of my own free will.**

※ Please listen to the interviewer's sufficient explanation for each survey item below, understand it, and then mark √ in the □ of the items you agree to participate in. ※ To participate in the research, you must agree to items ①, ②, and ③ among the collection items below to be recognized as a subject.

| **Collection Items** | Consent |
| --- | --- |
| \| 1. Survey: Demographic characteristics, health-related information, lifestyle habits, family history, occupational history, digital literacy, service model satisfaction, etc. \|  \| \| --- \| --- \| \|  \|  \| | □ |
| 1. Chronic disease-related medical records and test results | □ |
| 1. Lifelog data: Diet, physical activity, medication, self-measured blood pressure and blood sugar, etc. | □ |

**2.** I agree to the use of my data linked with public institutions' data for the purpose of confirming my health status.

| **Public Institution** | Data Content | Consent |
| --- | --- | --- |
| National Health Insurance Service | Health screening data - General health screening data, Medical use data - Health insurance qualification DB (age, region, income quintile, subscriber classification, etc.) | □ Agree  □ Disagree |

**※** The collected data will be utilized after being processed to be unidentifiable.

**3. I agree that the information provided to the research team will be provided to the research team and third parties (National Health Smart Management R&D Project Group under the Ministry of Health and Welfare).**

It will be submitted to the data platform (big data and personal data repository) built by this project group.

In the process of submitting data, personal information will be protected by applying the integrated authentication system and DID (Decentralized Identifier, blockchain technology-based distributed identity verification technology) system developed by this project group.

**[Purpose of Collection and Use]** ① Development and demonstration of a community-based chronic disease (hypertension and diabetes) management service model using digital health technology. ② Maintenance and management of research participants, mailing, etc.

**[Personal Information Collection Items]** Name, phone number, address, email, date of birth □ I agree to the collection and use of the above personal information.

**[Sensitive Information Collection Items]** Demographic characteristics, health-related information, lifestyle habits, family history, occupational history, digital literacy, etc. survey, medical records and clinical test results, lifelog data such as diet, physical activity, medication, self-measured blood pressure and blood sugar, etc. □ I agree to the collection and use of the above personal information.

**[Matters Concerning the Provision of Personal Information to Third Parties]** The collected personal information will be processed within the scope specified in the "Purpose of Processing Personal Information" and will not be processed beyond its original scope or provided to third parties without the user's prior consent. However, it will be processed as an exception only in cases where there are special provisions in other laws or in cases falling under Article 18 of the Personal Information Protection Act, such as criminal investigations.

**[Retention and Use Period]** The above personal information will be retained and used only for the period necessary to achieve the collection and use purposes. However, even if the purpose of collection and use has been achieved, personal information may be retained if there is a need for storage under other laws.

**[Right of Refusal and Disadvantages]** We inform you that you can refuse to participate in this survey at any time if you do not wish to, and there will be no disadvantages if you refuse.

- **I agree to the collection and use of personal information as above in accordance with the Personal Information Protection Act based on Articles 3 and 5 of the Personal Information Protection Act.**

**20 (Year)/ (Month)/ (date)**

|  |  |  |  |  |
| --- | --- | --- | --- | --- |
| Research Subject's Name |  | Signature |  | Date(Year/Month/Day) |
|  |  |  |  |  |
| Researcher's Name |  | Signature |  | Date(Year/Month/Day) |
|  |  |  |  |  |
| Legal Representative's Name |  | Signature |  | Date(Year/Month/Day) |
|  |  |  |  |  |
| (Relationship between subject and representative) |  | (Reason for representative consent) |  |  |
